# Epigenetic Regulation of Autophagy in Bone Metabolism

**DOI:** 10.1093/function/zqae004

**Published:** 2024-01-27

**Authors:** Yazhou Zhang, Qianqian Wang, Hongjia Xue, Yujin Guo, Shanshan Wei, Fengfeng Li, Linqiang Gong, Weiliang Pan, Pei Jiang

**Affiliations:** Department of Foot and Ankle Surgery, Tengzhou Central People’s Hospital, Tengzhou 277500, China; Department of Pediatric Intensive Care Unit, Tengzhou Central People’s Hospital, Tengzhou 277500, China; Department of Computer Science, University College London, London, WC1E 6BT, UK; Institute of Clinical Pharmacy & Pharmacology, Jining First People’s Hospital, Jining 272000, China; Department of Pharmacy, Shandong Provincial Hospital Affiliated to Shandong First Medical University, Jinan 250000, China; Department of Graduate, Shandong Academy of Medical Sciences, Shandong First Medical University, Jinan 250000, China; Department of Neurosurgery, Tengzhou Central People’s Hospital, Tengzhou 277500, China; Department of Gastroenterology, Tengzhou Central People's Hospital, Tengzhou 277500, China; Department of Foot and Ankle Surgery, Tengzhou Central People’s Hospital, Tengzhou 277500, China; Translational Pharmaceutical Laboratory, Jining First People’s Hospital, Shandong First Medical University, Jining 272000, China; Institute of Translational Pharmacy, Jining Medical Research Academy, Jining 272000, China

**Keywords:** epigenetics, autophagy, bone homeostasis, bone metabolism, miRNA, bone-related diseases

## Abstract

The skeletal system is crucial for supporting bodily functions, protecting vital organs, facilitating hematopoiesis, and storing essential minerals. Skeletal homeostasis, which includes aspects such as bone density, structural integrity, and regenerative processes, is essential for normal skeletal function. Autophagy, an intricate intracellular mechanism for degrading and recycling cellular components, plays a multifaceted role in bone metabolism. It involves sequestering cellular waste, damaged proteins, and organelles within autophagosomes, which are then degraded and recycled. Autophagy’s impact on bone health varies depending on factors such as regulation, cell type, environmental cues, and physiological context. Despite being traditionally considered a cytoplasmic process, autophagy is subject to transcriptional and epigenetic regulation within the nucleus. However, the precise influence of epigenetic regulation, including DNA methylation, histone modifications, and non-coding RNA expression, on cellular fate remains incompletely understood. The interplay between autophagy and epigenetic modifications adds complexity to bone cell regulation. This article provides an in-depth exploration of the intricate interplay between these two regulatory paradigms, with a focus on the epigenetic control of autophagy in bone metabolism. Such an understanding enhances our knowledge of bone metabolism-related disorders and offers insights for the development of targeted therapeutic strategies.

## Graphical Abstract

**Graphical Abstract:**
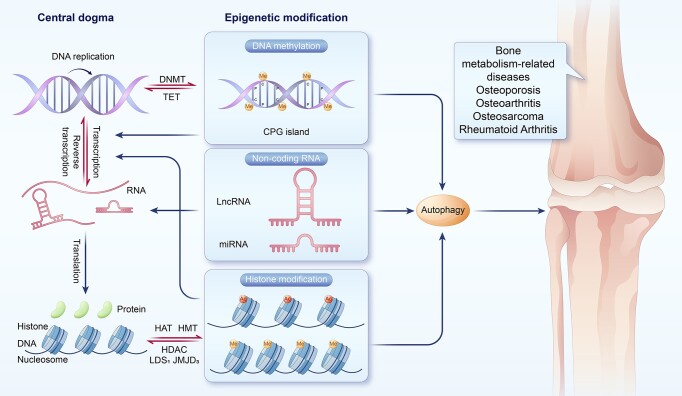

## Introduction

The skeletal system, characterized by its dense and robust tissues, serves as a cornerstone in supporting bodily functions, safeguarding vital organs, facilitating hematopoiesis, and storing essential minerals.^1–3^ Maintaining bone homeostasis is paramount for ensuring the normal functionality of the skeleton, encompassing critical aspects such as bone density, structural integrity, and regenerative processes.^4^

Autophagy sequesters cellular waste, damaged proteins, and organelles within autophagosomes, subsequently subject to degradation and recycling.^5^ The outcome of autophagy in bone metabolism depends on various factors, including autophagy regulation, cell type, environmental cues, and physiological context.^6^

While traditionally perceived as a cytoplasmic process,^7^ autophagy is subject to transcriptional and epigenetic regulation within the nucleus.^8–10^ However, the precise impact of epigenetic regulation, encompassing DNA methylation, histone modifications, and non-coding RNA (ncRNA) expression, on cellular fate remains incompletely elucidated.^8^ The interplay between autophagy and epigenetic modifications adds a layer of complexity to bone cell regulation.

This article explores the intricate interplay between these two regulatory paradigms, shedding light on the epigenetic control of autophagy within the realm of bone metabolism. This exploration enhances our comprehension of bone metabolism-related disorders and pondered the future and challenges of epigenetic regulation of autophagy in bone metabolism.

## Overview of Epigenetic Modifications

Epigenetics broadly pertains to inheritable modifications in phenotype that do not involve modifications in DNA sequences.^11,12^ While the genotype remains unchanged, hereditary alterations occur in the phenotype. Among the most well-established epigenetic mechanisms are DNA methylation, post-translational modifications of histones, and ncRNAs.^13–15^ These mechanisms serve as key regulators of gene expression, functioning either through the modulation of chromatin structure and gene transcription or, in the case of ncRNAs, through post-transcriptional control of protein translation.^16,17^ Epigenetics plays a regulatory role in various biological processes, including the specific expression of genes in different tissues, the inactivation of chromosomes, genomic imprinting, and the differentiation of cells.^18,19^ With the advancement of research techniques, epigenetic abnormalities have been implicated in causing malignancies, metabolic disorders, somatic diseases, and autoimmune diseases.^20^ Epigenetics highlights the intricate interplay between genetic and environmental factors and plays a crucial role in regulating bone metabolism,^21^ particularly in the differentiation of bone cells, providing insights into the study of bone metabolic disorders and their treatment directions.

### DNA Methylation

DNA methylation stands as one of the most extensively studied and common epigenetic modifications in mammals.^22^ This entails methylating the 5′ carbon of cytosine, leading to the formation of 5-methylcytosine (5-mC).^23,24^ Cytosine methylation predominantly occurs within specific dinucleotide sequences known as CpG sites. Importantly, these CpG sites are not randomly distributed across the genome but are concentrated in regions called CpG islands, often situated at the 5′ ends of genes. High methylation levels in CpG islands are commonly associated with gene silencing, while low methylation levels are linked to gene activation.^25,26^

The enzymes responsible for transferring methyl groups from *S*-adenosylmethionine (SAM) to DNA include DNA methyltransferase 1 (DNMT1), DNMT3a, DNMT3b, and DNMT3L.^27^ Demethylation of DNA can occur either passively following DNA replication, leading to reduced maintenance methylation, or actively.^28^ The precise molecular mechanisms underlying active demethylation are not yet fully understood. For instance, in the context of bone biology, Interferon Regulatory Factor 8 (IRF8) transcription factor inhibits osteoclast formation.^29^ Experimental evidence has revealed that DNMT3a plays a significant role in inhibiting the activity of IRF8 by increasing methylation at distal regulatory elements associated with the *IRF8* gene. Elevated levels of SAM can enhance this methylation, ultimately promoting osteoclast differentiation and bone resorption.^30^ Deleting DNMT3a in osteoclasts (OC) or using the inhibitor TF-3 in mice protects against bone loss after ovarian removal.^31^ Moreover, in multiple myeloma patients, alterations in bone resorption have been associated with elevated IRF8 methylation, induced by myeloma cells’ release of thymidine phosphorylase (TP), leading to decreased *IRF8* expression and enhanced bone resorption.^32^

The transcription factors runt-related transcription factor 2 (Runx2) and Osterix (OSX) play critical roles in regulating osteoblast (OB) differentiation and bone matrix synthesis. Runx2 activates the promoter of OSX, indicating Runx2’s position as an upstream transcription factor in osteogenesis.^33^ During mesenchymal stem cell differentiation into osteoblasts, there is a reduction in the methylation level of Runx2, underscoring the significant regulatory function of Runx2 methylation during osteoblast differentiation.^29^ Additionally, epigenetic regulation of OSX is *vital* for guiding mesenchymal stem cells (MSCs) into osteoblast differentiation.^34^

The Ten-Eleven-Translocation (TET) family of enzymes are recognized for their ability to remove methyl groups from DNA, converting 5-mC into 5-hydroxymethylcytosine (5-hmC). Demethylation mediated by TET enzymes enhances chromatin accessibility for target genes controlled by Runx2, facilitating transcriptional regulation. TET proteins engage with Runx2 *via* their catalytic domain, influencing cytosine methylation patterns around the Runx2 binding region.^35^ Disruptions in the promoter methylation of key bone-related genes, such as bone morphogenetic protein 2 (BMP2), may lead to irregularities in bone formation.^29^

### Post-Translational Histone Modifications

Inside the eukaryotic nucleus, a segment of double-stranded DNA spanning 147 base pairs coils around eight histone proteins, which include two copies of H2A, H2B, H3, and H4, creating nucleosomes. These nucleosomes are linked by DNA segments and organize into structured chromatin formations called chromatin, which subsequently compact into chromosomes.^36–38^ The N-terminal amino acid tails of H3 and H4 undergo post-translational modifications that profoundly affect both chromatin structure and DNA-associated activities.^39^ Research has unveiled that euchromatin, representing a relaxed and actively transcribed DNA state, is marked by elevated levels of acetylation and trimethylation at H3K4, H3K36, and H3K79. In contrast, reduced acetylation levels and heightened methylation at H3K9, H3K27, and H4K20 indicate a more condensed, transcriptionally inactive heterochromatin structure.^23,40^ Histone modification is the most intricate mode of regulation among the three epigenetic modifications.^41^ Currently, the most extensively studied histone modifications in bone metabolism are histone acetylation and histone methylation.

#### Histone Acetylation

Histone acetylation involves the addition of acetyl groups to histones and is governed by histone acetyltransferases (HATs). Conversely, histone deacetylases (HDACs) regulate the removal of these acetyl groups, leading to histone deacetylation.^42^ This balance between HATs and HDACs maintains a delicate equilibrium.

Histone acetyltransferases facilitate the attachment of acetyl-CoA molecules onto histones, promoting a relaxed nucleosome structure.^43^ This structural change activates the transcriptional machinery, enhancing gene expression. In cases where HAT activity is hindered or inhibited, the repair of damaged DNA may be compromised, potentially resulting in cellular apoptosis or programmed cell death. This underscores the crucial role of HATs in DNA repair and cell survival.^44^ Unlike HATs, HDACs remove acetyl groups from histones, causing histones to tightly bind to negatively charged DNA. This leads to dense chromatin compaction and the inhibition of gene transcription.^45^

Histone acetyltransferases are classified into distinct subfamilies based on their catalytic domains structural and functional similarities, represented as HAT domains. These subfamilies include p300/CBP, HAT1, MYST, PCAF/Gcn5, and Rtt109.^46^ Conversely, HDACs are categorized into 4 classical classes according to their sequence similarities: Class I, Class II, Class III, and Class IV.^47^ The Class I, II, and IV HDACs contain a conventional deacetylase domain, while Class III HDACs feature a NAD^+^-dependent catalytic domain (Table 1).^48^

**Table 1.** Histone acetyltransferase, histone deacetylase, and their role in bone metabolism.

HATs/HDACs	Target genes	Function	Reference
P300/CBP	Runx2	Promotes the transcriptional activity of Runx2 to promote osteogenic differentiation	^50^
	NFATC1	RANKL promotes osteoclast differentiation through NFATc1 acetylation	^61^
PCAF	Runx2	Promotes osteoblast differentiation	^62^
	CXCL12	Promotes osteogenic differentiation of MSCs	^63^
	NFATC1	Promotes osteogenic differentiation of MSCs	^61^
GCN5	Wnt, NF-kB	Enhances osteogenic differentiation ability of BMSCs	^64,65^
HDAC1	FoxO3a	Promotes MSC osteogenic differentiation	^53^
HDAC4		HDAC4 acts as an oncogene in osteosarcoma cancer to promote cell proliferation and inhibit apoptosis and autophagy	^66^
	Runx2	Deacetylates and degrades Runx2, leading to reduced osteoblast function	^67^
SIRT1	Runx2	Promotes osteogenic differentiation of MSCs	^68^
	FoxO3	Reduces FoxO acetylation levels in BMMs, scavenges ROS, and inhibits bone resorption	^69,70^

The transcriptional activity of the *Runx2* gene is modulated by the acetyltransferase P300 and nicotinamide phosphoribosyltransferase (Nampt). P300 enhances osteogenic differentiation in MSCs through H3K14 acetylation, while Nampt does so in MC3T3-E1 cells *via* H3K9 acetylation.^49,50^ Other acetyltransferases, GCN5 and PCAF, which acetylate histone H3K9, are known to enhance osteogenic differentiation and bone formation by acetylating H3K9 loci in the promoters of *Wnt* and *BMP* genes. However, their levels have been found to decline significantly in mice with ovariectomy-induced osteoporosis (OVX).^51,52^ In contrast to acetyltransferases, 2 HDACs, HDAC1 and Sirtuin1 (SIRT1), play roles in osteogenesis. HDAC1 promotes MSC osteogenesis through the deacetylation of Forkhead box O3a (FoxO3a), while SIRT1 inhibits osteogenic differentiation in Bone Marrow Stromal Cells (BMSCs) by deacetylating JAGGED1 (JAG1).^53,54^

In terms of bone health, numerous studies indicate that HDACs, particularly SIRT, play a significant role in bone development. They influence processes such as bone formation, repair, and regeneration.^55^ SIRT1, a representative of Class III HDACs, activation induces autophagy during cellular stress, directly deacetylating autophagy-related proteins (ATG5, ATG7, LC-3) to initiate autophagy.^56,57^ SIRT1 also deacetylates FoxO3, a transcription factor for autophagy-related genes.^58^ SIRT1 plays a pivotal role in driving MSC differentiation toward osteoblasts.^59,60^

#### Histone Methylation

Histone methylation mainly happens at lysine (K) and arginine (R) residues found at histones’ N-terminal regions. In contrast to acetylation, methylation events at these sites contribute to both transcriptional activation and inhibition. For instance, trimethylation of H3K4, H3K36, and H3K79 is associated with gene activation, while trimethylation of H3K27, H3K9, and H4K20 is linked to gene repression.^71,72^

Histone methylation is dynamically regulated by methyltransferases and demethylases.^73,74^ Methylation enzymes such as Suppressor of Variegation 3-9 Homolog 1 (SUV39H1), G9a, and Enhancer of Zeste Homolog 2 (EZH2) add methyl groups, while demethylation enzymes such as Lysine-specific demethylase 1 (LSD1) and JmjC domain (JMJD)-containing proteins remove methyl groups.

Methyltransferases and demethylases play pivotal roles in regulating gene expression in osteoblasts and osteoclasts, thereby influencing the functioning of related genes. (Table 2) For instance, SUV39H1/2 methyltransferases predominantly regulate the abundance of trimethylated H3K9. Knockdown of SUV39H1 leads to a reduction in H3K9me3 levels, enhancing DNA repair capabilities and delaying cellular senescence in progeroid cells.^75^

**Table 2.** Common histone methyltransferases and demethylases involved in histone methylation modification, as well as their target sites, genes, and their roles in bone metabolism.

Histone methyltransferases/ demethylases	Target histone sites	Target genes	Function	Reference
SUV39H1	H3K9me2/3	Runx2	Delays osteoblast differentiation	^83^
G9a	H3K9me2	Runx2	Regulates proliferation and differentiation of cranial bone cells	^84^
	H3K27me1	MMP-9	Induces expression of osteoclastogenesis-related genes and promotes osteoclast differentiation (24).	^85^
EZH2	H3K27me3	Wnt4, Foxo1	Enhances both osteogenesis and osteoclastogenesis	^86^
	H3K27me3	Wnt1, Wnt6, Wnt10a	BMMCs hinder bone formation and promote adipogenesis.	^77,78^
SETD2	H3K36me3	LBP	Guiding mesenchymal stem cells to commit to osteogenic destiny, diminishing their transformation into adipocytes	^79^
LSD1	H3K4me1	Runx2	Inhibits osteoblast differentiation of C2C12 cells	^87^
	H3K4me2	Wnt7b, BMP2	Inhibits osteogenic differentiation of BMSCs	^88^
JMJD2B (KDM4B)	H3K9me3	Runx2, CCND1	Promotes osteogenic differentiation of BMSCs and maintains bone-fat balance	^89,90^
JMJD3 (KDM6B)	H3K27me3	Runx2, OSX	Regulates osteoblast differentiation	^81^
		NFATC1	Promotes osteoclast differentiation	^80^

Excessive EZH2 activity leads to elevated H3K27me3 levels, causing a shift in the lineage commitment of BMMSCs toward adipocytes during osteoporosis.^76^ Further investigations reveal significant enrichment of both EZH2 and H3K27me3 in the promoters of Wnt1, Wnt6, and Wnt10a within BMMSCs of mice subjected to ovariectomy.^77,78^ Notably, EZH2 reduces the enrichment of H3K27me3 on these promoters, consequently suppressing the expression of *Wnt* genes. Overexpressing EZH2 results in heightened H3K27me3 levels at the transcription start sites (TSS) of Runx2 and Bglap, pivotal factors initiating osteogenesis.

The histone methyltransferase SET-domain-containing 2 (SETD2) catalyzes the modification of H3K36 trimethylation. It facilitates the binding of trimethylated histones to promoters associated with lipopolysaccharide-binding protein (LBP), thus influencing the specification of adipogenic and osteogenic pathways. Disrupting SETD2 through knockout shifts the fate of mesenchymal stem cells toward adipocyte formation, impairing their potential to differentiate into osteoblasts. In mice lacking Setd2 specifically in osteoprogenitor cells, there is a notable decrease in trabecular volume and bone formation rate, accompanied by an excessive accumulation of marrow fat.^79^

JMJD3 functions as a demethylase targeting H3K27. JMJD3 plays a regulatory role in influencing the expression of genes related to bone health, such as nuclear factor of activated T-cells cytoplasmic 1 (NFATC1),^80^ Runx2, OSX, osteopontin, bone sialoprotein (BSP), and osteocalcin (OCN).^79,81^  *In vitro* experiments reveal that inhibiting LSD1 through knockdown using shRNA or pharmacological inhibitors suppresses osteoblast function and differentiation. When LSD1 activity is inhibited in vivo, it leads to a reduction in both osteoblast count and activity, consequently resulting in osteopenia. Selective elimination of LSD1 from mesenchymal cells also results in osteopenia and disturbs the structure of the growth plate.^82^

### Non-Coding RNAs

Non-coding RNAs constitute a significant portion of the human genome, with approximately 2% consisting of protein-coding genes. The majority of the genome is made up of non-protein-coding RNAs,^91,92^ involving long non-coding RNAs (lncRNAs), circular RNAs (circRNAs), and small non-coding RNAs (sncRNAs). Within the category of sncRNAs, there are further subdivisions, such as microRNAs (miRNAs), piwi-interacting RNAs (piRNAs), small-interfering RNAs (siRNAs), and more. These ncRNAs play crucial roles in gene expression, cell differentiation, development, and various diseases, making them a prominent area of study in modern biology.^93^

#### Long Non-Coding RNAs

Long Non-Coding RNAs (LncRNAs) are ncRNAs that consist of more than 200 nucleotides in length. They have emerged as non-canonical regulators participating directly in various pathophysiological processes, including autophagy.^94^

As research advances, increasing evidence suggests that lncRNAs, as key regulators of gene expression, play pivotal roles in the proliferation, differentiation, apoptosis, and activity of osteoblasts and osteoclasts (Table 3). Disruptions in their expression patterns have been linked to numerous diseases, such as aging, cancer, metabolic disorders, and osteoporosis (OP).

**Table 3.** LncRNA participates in bone metabolism by functioning as a miRNA sponge or by modulating the activity of transcription factors and signaling pathways.

LncRNA	Bind miRNA	Target or signaling pathway	Effect on bone cell differentiation	References
PGC1β-OT1	miR-148a-3p	KDM6B	Promotes osteogenic differentiation	^95^
OGRU	miR-320-3p	Hoxa10 protein	Promotes osteoblast differentiation	^96^
AK0	—	PI3K/AKT	Regulates the proliferation of osteoblasts	^97^
Linc02349	miR-25-3p and miR-33b-5p	Smad 5, Wnt 10b	Promotes osteogenic differentiation	^98^
KCNQ1OT1	miR-701-3p	FGFR3	Promotes the proliferation, migration, and survival of osteoblasts	^99^
LOC100506178	miR-214-5p	BMP2	Promotes the differentiation of hBMSCs into osteoblasts	^112^
TUG1	miR-545-3p	CNR2	Promotes the proliferation and differentiation of osteoblast precursor cells hFOB1.19	^113^
GAS5	miR-135a-3p	FoxO1	Promotes osteoblast differentiation	^114^
MALAT1	miR-30	Runx2	Promotes Osteoblast Differentiation of hADMSCs	^100^
Rhno1	miR-6979-5p	BMP2	Promotes osteogenic differentiation	^101^
MCF2L-AS1	miR-33a	Runx2	Stimulates osteogenic differentiation in hBMSCs	^102^
H19	miR-149	SDF-1	Promotes osteoblast differentiation	^103^
MEG3	—	BMP4	Promotes the osteogenic differentiation of MSCs	^115^
H19	miR-185-5p	IGF-1	Promotes mineralization in osteoblasts	^116^
PRNCR1	miR-211p-5p	CXCR4	Inhibits osteogenic differentiation	^117^
ANCER	—	Wnt	Inhibits the osteogenic differentiation of hPLSCs	^118^
HOTAIR	—	Wnt*/*β-catenin	Inhibits osteogenic differentiation of BMSCs	^105^
ODIR1	—	FBXO25/H2BK120ub/ H3K4me3/OSX Axis	Inhibits osteogenic differentiation of HUC-MSCs	^106^
SNHG1	miR-101	—	Inhibits osteogenesis differentiation	^107^
UCA1	—	BPM2/Smad1/5/8	Inhibits osteoblast proliferation and differentiation	^108^
MIRG	miR-1897	NFATc1	Promotes osteoclast production	^109^
NEAT1	miR-7	PTK2	Increases expression of osteoclast marker genes	^110^
Bmncr	—	RANKL	Inhibits osteoclast differentiation	^119^
NRON	—	NFATC1	Inhibits osteoclast differentiation	^111^

Several lncRNAs have been identified as positive regulators of osteoblastogenesis, including PGC1β-OT1,^95^ OGRU,^96^ AK0,^97^ LINC02349,^98^ KCNQ10T1,^99^ MALAT1,^100^ Rhno1,^101^ MCF2L-AS1,^102^ H19,^103^ GAS5,^104^ and more. These lncRNAs promote osteogenic differentiation by various mechanisms, such as acting as sponges for miRNAs or regulating key osteogenic factors. On the other hand, some lncRNAs negatively regulate osteogenic differentiation, including HOTAIR,^105^ ODIR1,^106^ SNHG1,^107^ UCA1,^108^ and more. These lncRNAs inhibit osteogenesis by suppressing specific signaling pathways or inhibiting the expression of osteogenic marker genes.

Long non-coding RNAs also play dual roles in osteoclastogenesis. For example, MIRG has been shown to have a positive regulatory role,^109^ while NEAT1^110^, Bmncr,^110^ NRON,^111^ and others inhibit osteoclast formation by various mechanisms. Overall, lncRNAs represent a diverse class of regulatory molecules with complex roles in the regulation of osteoblasts and osteoclasts, influencing bone health and associated diseases.

##### MicroRNAs

MicroRNAs (miRNAs) are short, ncRNA molecules typically composed of 19 to 25 nucleotides in length. They are highly conserved across species and play a crucial role in post-transcriptional gene regulation.^120^ MicroRNAs do not code for proteins themselves but instead regulate gene expression by binding to complementary sequences within the target mRNA, leading to the degradation of the target mRNA or translational repression.^121^ The intricate interplay between miRNAs and their target mRNAs is influenced by various factors, including the strength of their interaction, target mRNA abundance, and intracellular localization of both miRNA and mRNA.^122^

In the context of osteogenesis and bone cell differentiation, miRNAs have several key roles: regulation of bone formation, diverse functions and mechanisms, targeting multiple genes and potential therapeutic targets (Table 4).

**Table 4.** The role of miRNA in osteogenesis and bone cell differentiation.

Function	Functional characteristics	Phenotype	Reference
Regulation of bone formation	Regulates osteogenic differentiation signaling pathways	MiR-335-5p promotes bone formation and regeneration by activating Wnt signaling and specifically downregulating the expression of DKK1	^123^
		MiR-196a promotes hASC osteogenic differentiation through BMP/Smad signaling pathway	^124^
		MiR-210 positively regulates osteoblast differentiation through TGF-β/activin signaling pathway	^125^
Diverse functions and mechanisms	A single miRNA can serve multiple functions within bone cells, and they operate through various mechanisms	MiR-21, miR-140 and miR-214 respectively affect fracture healing through multiple mechanisms and have been identified as potential biomarkers of fracture healing.	^126^
	Engage in complex signaling networks, including both feedforward and feedback mechanisms, to modulate the functionality of bone cells	MiR-21 regulates osteoclastogenesis through the positive feedback loop of c-Fos/miR-21/PDCD4	^127^
		MiR-125a regulates osteoclast differentiation through a TRAF6/NFATc1/miR-125a negative feedback loop	^128^
Targeting multiple genes	MiRNAs have multifaceted effects on bone cell differentiation by targeting multiple genes	The miR-29 family is involved in bone cell differentiation, with individual miRNA members targeting different genes. miR-29b suppresses Secreted Frizzled-Related Protein 1 (SFRP1) and collagen expression in mature osteoblasts, while miR-29a targets Dickkopf-Related Protein 1 (DKK1) and bone sialoprotein.	^129^
		MiR-422a is up-regulated in osteoporosis, and it simultaneously inhibits 5 genes: *CBL, CD226*, IGF1, *PAG1*, and *TOB2*	^130^
Potential therapeutic targets	MiRNAs may be concentrated in cell populations associated with functions linked to specific cell phenotypes, making them potential drug targets (miR-146a, miR-29b, miR-124 possible target for drugs to treat osteoporosis)	MiR-146a inhibits TNF-a/RANKL-induced osteoclastogenesis in human PBMCs *via TRAF6*	^131^
		MiR-29b inhibits M-CSF and RANKL-induced osteoclastogenesis in CD14 hematopoietic stem cells via *c-Fos*	^132^
		MiR-124 modulates osteoclast formation in mouse bone marrow macrophages (BMMs) by inhibiting NFATc1 expression.	^133^

Overall, miRNAs are essential regulators of osteogenesis and bone cell differentiation, contributing to the maintenance of bone tissue and the equilibrium between bone formation and resorption. Understanding the roles and regulatory networks of miRNAs in bone biology is crucial for advancing our knowledge of bone-related diseases and potential therapeutic interventions.

## Autophagy in Bone Metabolism

In mammals, three specific types of autophagy have been recognized: macroautophagy, microautophagy, and chaperone-mediated autophagy. Of these, macroautophagy is the most prevalent and is intricately intertwined with cellular physiology, biological functions, and the development of diseases within the context of bone.^134^ In this review, we will primarily focus on macroautophagy when referring to autophagy.

Autophagy typically progresses through four key stages: the creation of autophagosome precursors, followed by the formation of autophagosomes, then the development of autolysosomes, and finally, the degradation phase. During the degradation phase, sizable cellular molecules are disintegrated into amino acids, lipids, nucleotides, and energy, facilitating both the cell's metabolic necessities and the rejuvenation of specific organelles.^135^

Currently, there are more than 40 ATG genes known to regulate autophagy.^136^ Each gene plays distinct roles at specific stages. ULK1, a key protein, forms the ULK1 complex (ULK1-ATG13-FIP200-ATG101) initiating autophagy. During starvation, mTOR inhibition and AMPK activation prompt ULK1 phosphorylation, initiating autophagy. Beclin1, similar to yeast ATG6, interacts with proteins to form the PI3K complex, crucial for autophagy initiation. ATG14 and UVRAG play roles in autophagosome elongation and autophagosome-lysosome fusion, respectively. Proteins such as ATG12, ATG5, and LC-3 control autophagosome formation. LC3-II, a key autophagy marker, encapsulates materials for degradation. Finally, the autophagosome fuses with lysosomes to form autolysosomes, where degradation provides cells with energy against stress.^137^

### Importance of Autophagy in Bone Homeostasis and Remodeling

**Figure 1.**
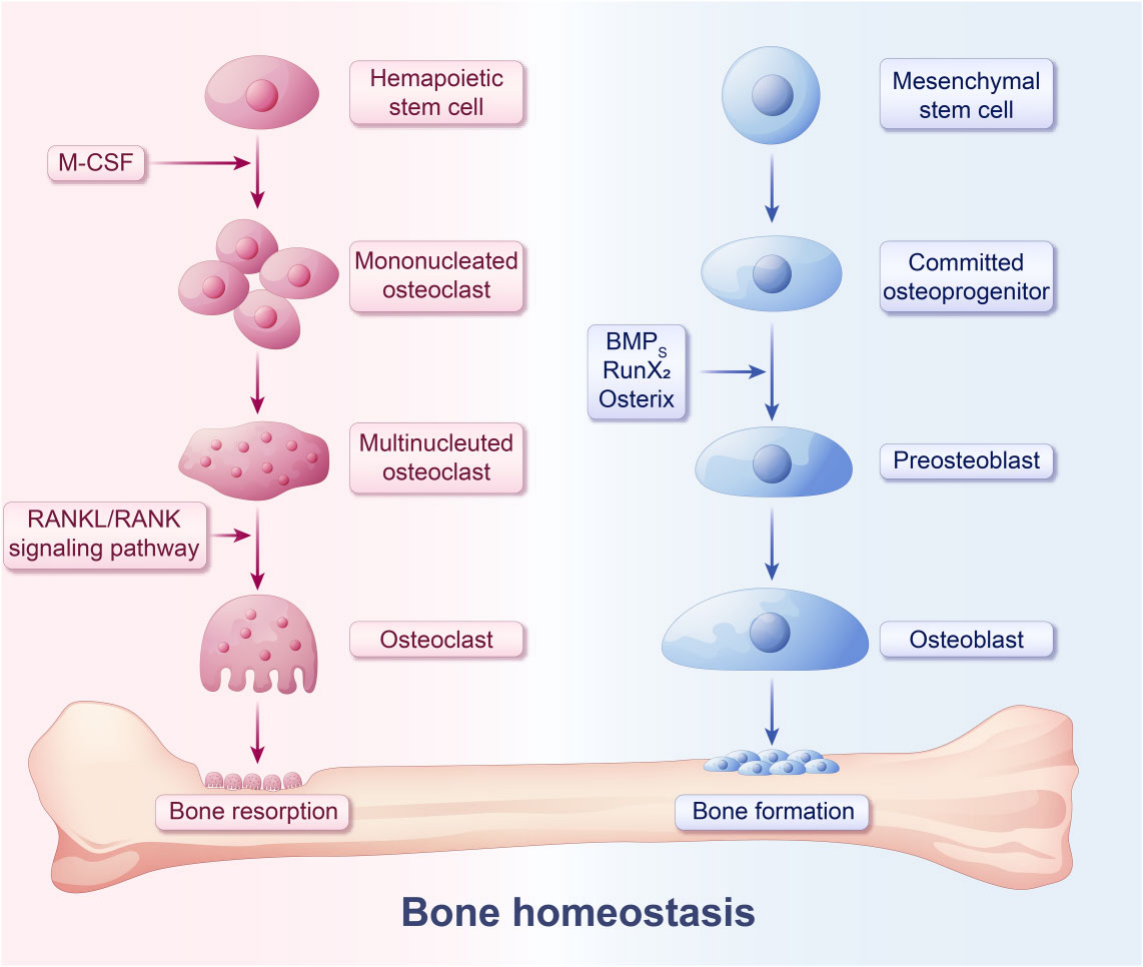
Bone resorption and bone formation maintain the dynamic balance of bone homeostasis. Mesenchymal stem cells in the bone marrow gradually differentiate into osteoblasts, with key transcription factors such as BMPS, Runx2, and OSX playing critical roles in regulating osteoblast differentiation and bone matrix synthesis. Hematopoietic stem cells differentiate into mononucleated osteoclasts under the influence of M-CSF secreted by osteoblasts. Upon activation by RANKL-RANK signaling, these cells further differentiate into mononuclear resorbing cells and subsequently fuse into multinucleated osteoclasts.

Bone homeostasis refers to the delicate balance maintained within the skeletal system through a dynamic interplay between bone-forming cells (osteoblasts), bone-resorbing cells (osteoclasts), and bone matrix.^138^ This intricate equilibrium is crucial for preserving the overall health, strength, and functionality of bones (Figure 1).

Bone remodeling involves three cell types: MSCs differentiate into osteoblasts (OB) on the bone surface, secreting bone matrix. This matrix, with OB, transforms into osteocytes, forming a vital mechanosensory network in bones, essential for signaling; simultaneously, multinucleated osteoclasts (OC) derived from hematopoietic stem cells constantly break down and absorb the neighboring bone matrix.^138,139^ Generally, the balance between bone formation and resorption is continually coordinated. In this manner, the quality, structure, and function of bone tissue can be influenced by internal or external stimuli. Autophagy helps OB, OC, and chondrocytes cope with stress and nutrient deficiencies, promoting survival in harsh hypoxic and hypertonic conditions. Autophagy can also enable the long-term terminal differentiation of osteocytes. The process of autophagy encompasses not only the osteoclastic resorption process but also the acquisition of energy sources during osteoblast differentiation.^140^ Altered autophagy can disrupt bone cell balance, potentially causing various diseases.

### Role of Autophagy in Mesenchymal Stem Cells, Osteoblasts, Chondrocytes, Osteoclasts, and Osteoclasts

#### Autophagy in BMSCs

As a rare and diverse subset of stromal cells, BMSCs exhibit the capacity for both self-renewal and differentiation. These cells are capable of undergoing differentiation into various lineages, not limited solely to mesenchymal lineages such as osteocytes, chondrocytes, and adipocytes,^141,142^ autophagy is vital in controlling the roles, differentiation, and survival of BMSCs. Modulating autophagic activity in these stem cells could have implications for enhancing bone regeneration, treating bone-related diseases, and addressing age-related bone health issues.

Autophagy-regulated redox state participates in determining the fate of BMSC differentiation. It has been reported that elevated ROS levels in BMSCs can promote adipogenesis while inhibiting osteogenic differentiation. When excessive ROS is generated within cells, the autophagic mechanism is activated to reduce ROS levels, thereby restoring osteogenic differentiation of BMSCs.^143^ Additionally, research has demonstrated that administering the neuropeptide substance P (SP) to rats enhances BMSC autophagic activity through the AMPK and mammalian target of rapamycin (mTOR) pathways, concurrently reducing ROS production and facilitating osteogenic differentiation.^144^ Rapamycin (RAPA), a well-known inhibitor of the mTOR, triggers autophagy by binding to mTOR and activating the mTOR signaling pathway. Research has indicated that RAPA enhances autophagy and influences the osteogenic differentiation of MSCs.^145^

#### Autophagy in Osteoblast

Osteoblasts are central to the processes of bone growth, repair, and remodeling. They synthesize collagen proteins and other bone matrix molecules, providing structural support to the skeleton. Osteoblasts also release bone formation-related proteins, hormones, and cytokines, such as alkaline phosphatase, osteocalcin, and others, which play significant roles in regulating bone metabolism and maintaining bone homeostasis.^146^

Autophagic proteins such as Beclin1, ATG5, and ATG7 play an essential role in facilitating the mineralization of osteoblast cell lines. Osteoblast autophagy deficiency can reduce its mineralization ability, leading to a low bone mass phenotype.^147^ Insufficient or deficient autophagy in osteoblasts leads to an increase in oxidative stress, which in turn elevates the production of TNFSF11. This further enhances the differentiation of osteoclasts, ultimately resulting in a phenotype resembling osteoporosis.^148^ Transcription factors FoxO and ATF4 have been extensively studied in the regulation of autophagy during the differentiation and function of osteoblasts. FoxO binds to the promoter regions of autophagy genes, increasing autophagic activity. This activation may promote the differentiation of MSCs into osteoblasts and inhibit fat generation.^149^ Additionally, under endoplasmic reticulum (ER) stress and amino acid deficiency conditions, ATF4 can promote autophagosome formation and autophagic flux by regulating the expression of autophagy initiation-related genes, contributing to the maintenance of osteoblast homeostasis.^150^

#### Autophagy in Osteoclasts

Osteoclasts primarily engage in bone resorption in the body and play a role in bone homeostasis. When their activity is excessively high, it can lead to osteoporosis, while conversely, decreased activity can result in increased bone formation.^151^ The microenvironment where osteoclasts are distributed, such as the sealing zone and the interior of bone trabeculae, is characterized by low oxygen levels, which support the survival and maturation of osteoclasts.^152^ Under low oxygen conditions, the expression of Hypoxia-inducible factor 1 alpha (HIF-1α) and its downstream signaling molecule BNIP3 increases, leading to elevated levels of autophagy-related proteins such as ATG5, ATG12, and Beclin1. Consequently, LC-3 is recruited to autophagosomes, enhancing the expression of bone resorption factors such as RANKL, matrix metalloproteinase K (MMP), tissue protease K, NFATc1. This, in turn, leads to increased osteoclast formation.^153,154^ Research has provided evidence that the targeted removal of ATG7 in osteoclast precursors in mice resulted in the improvement of bone loss and the excessive activation of osteoclasts triggered by glucocorticoids or ovariectomy.^155^

During the adhesion and migration of osteoclast precursor cells, chemotactic factors CXCL12 and S1P play roles in this process. Specifically, S1P binds to its receptor S1PR on the membrane of osteoclast precursor cells, participating in the migration process.^156^ S1P also modulates autophagy through mTOR, serving as a link between autophagy and the accumulation of osteoclasts.^157^ RANKL and RANK (receptor activator of NF-κB) play essential roles in osteoclast differentiation and maturation. RANKL induces autophagy activation through pathways such as MAPK and NF-κB during this process.^158^

#### Autophagy in Osteocytes

Osteocytes establish an extensive interconnected network throughout the entire skeleton. They do this through multiple branching processes that resemble dendrites, allowing them to connect with other types of bone cells such as osteoblasts, bone lining cells, and stromal cells.^159^ This network spans from the innermost bone regions to the blood vessel linings.^160^ Osteocytes play multifaceted roles: they regulate mineral metabolism and the remodeling of the perilacunar matrix, while also serving as mechanosensory cells.^161^

Osteocytes play a central role in regulating bone remodeling in response to mechanical loading.^162^ Autophagy is a vital mechanism that ensures the survival of osteocytes and enhances their capacity for mechanotransduction in the context of bone remodeling. Recent investigations have unveiled autophagy’s responsiveness to mechanical cues in osteocytes. Mechanistically induced autophagy contributes to the preservation of adenosine triphosphate (ATP) and fosters osteocyte survival. Furthermore, given the unique features of terminal differentiation, the prolonged lifespan of bone cells, and their oxygen and nutrient-deprived environment, autophagy becomes a key player in the regular physiological processes of these cells.^140,163^ Unsurprisingly, osteocytes exhibit a notable baseline level of autophagy both in laboratory settings and within living organisms. Selectively deleting the autophagy-related gene *ATG7* in osteocytes inhibits autophagy, causing decreased bone formation and reduced bone mass in young adult mice, resembling the effects of aging on the skeletal system.^164^ Dysregulated autophagy in osteocytes, as seen in Ephrin B2 deficiency or triggered by substances such as pinocembrin, can affect bone health and apoptotic processes.^165,166^ Beyond its role as a degradative mechanism, recent evidence has spotlighted the involvement of autophagy in protein secretion, referred to as secretory autophagy, bridging intracellular autophagy with the extracellular microenvironment.^167^ This phenomenon may offer insights into understanding the impacts of osteocyte autophagy triggered by physical forces exerted on osteoblasts and osteoclasts.^168^

#### Autophagy in Chondrocytes

MSCs in the bone marrow differentiate into osteoprogenitor cells, which further differentiate into chondrocytes that form the cartilage primordia.^169,170^ Conversely, they develop into endochondral bone, determining the rate and length of longitudinal bone growth. Most skeletal growth is achieved through the ossification of cartilage in the epiphyseal growth plate of bones. Due to the low regenerative capacity of chondrocytes and limited vascularity within the growth plate, chondrocytes are prone to hypoxia and nutrient deficiency.^171^ It has been demonstrated that inflammatory mediators including reactive oxygen species (ROS), IL-1β, nitric oxide (NO), Fas, and tumor necrosis factor-alpha (TNF-α) are strongly associated with chondrocyte apoptosis.^172^

Autophagy, a cellular degradation mechanism responsible for maintaining cellular energy metabolism homeostasis, possesses the ability to restore impaired chondrocyte functionality.^173^ When cellular ATP levels decrease, AMPK activates and triggers autophagy, restoring nutrients and ATP, thus maintaining cellular energy balance. Autophagy also impacts protein and lipid metabolism in chondrocytes through the mTOR pathway.^174^

It has been demonstrated that chondrocyte degeneration and apoptosis are considered primary factors in the development of osteoarthritis (OA).^175^ Consequently, numerous experiments have been designed to enhance chondrocyte autophagy as a means to alleviate OA. For instance, Interventions such as vitamin D and tofacitinib exhibit potential in protecting against OA by promoting autophagy and preventing chondrocyte degeneration.^176,177^ Proteins such as PGRN and G protein-coupled receptor family C group 5 member B (GPRC5B) play roles in maintaining chondrocyte health through their involvement in autophagy.^178,179^ Moderate mechanical strain can promote the restoration of metabolic homeostasis by inhibiting inflammation and excessive autophagy.

## Epigenetic Regulation of Autophagy in Bone Metabolism

Epigenetic modifications, alterations in gene expression without DNA sequence changes,^11^ intersect significantly with autophagy, a process vital for cellular stress response and recycling. This intricate relationship profoundly influences bone metabolism and homeostasis.

### Epigenetic Modifications and Autophagy

DNA methylation, a heritable epigenetic modification, impacts autophagy regulation in diverse cancers and stem cells.^173^ Hypermethylation of LC-3A^174^ and Beclin1^175^ inhibits autophagy, promoting tumorigenesis. Conversely, demethylation enhances autophagy, showing therapeutic potential in cancer treatments.^176,178,179^ Additionally, in conditions like osteoporosis and osteoarthritis, abnormal DNA methylation affects autophagy, highlighting its role in bone health.^180^

Histone modifications such as methylation and acetylation regulate autophagy-related genes. G9a-mediated histone methylation inhibits autophagy, while its dissociation activates this process, crucial in non-alcoholic fatty liver disease.^181^ Enzymes such as JMJD3^182,183^ and SIRT^184,185^ family members modulate histone demethylation and deacetylation, respectively, impacting autophagy regulation in cellular processes.

Non-coding RNAs, including miRNAs and lncRNAs, regulate autophagy at different stages in various diseases.^186^ MiRNAs influence autophagy-related genes, affecting cancer cell survival and response to treatments.^187–190^ Long non-coding RNAs, such as H19^191,192^ and GAS5,^193,194^ exhibit both positive and negative regulatory effects on autophagy, impacting bone-related disorders such as osteoarthritis.

### Epigenetic Regulation of Autophagy in Bone Metabolism-Related Diseases

**Figure 2.**
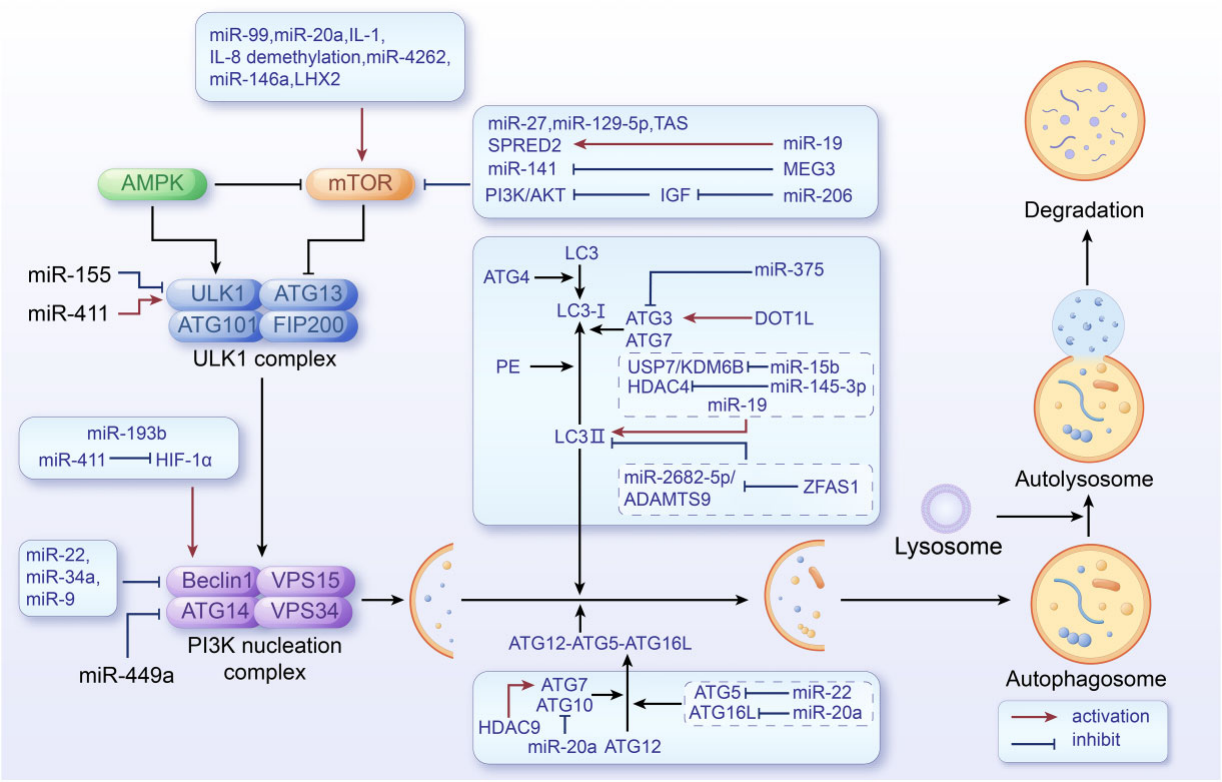
Under the influence of genetic or pathogenic environmental conditions, histone modifications and ncRNAs promote or inhibit autophagy by targeting autophagy-related genes and proteins. Disordered autophagy leads to the occurrence and progression of bone metabolism-related diseases.

Epigenetic mechanisms are crucial in regulating autophagy processes, influencing its transcriptional and post-translational regulation.^195,196^ These epigenetic mechanisms can be further influenced by external stimuli, alterations in phenotypic states, or pathological environmental conditions.^197^ Epigenetic modifications and autophagy interact in bone metabolism, regulating gene expression, cellular clearance, and stress responses, crucial for maintaining bone tissue homeostasis and function. The convergence of genetic and environmental factors, causing disruption in the epigenetic regulation of autophagy, has the potential to detrimentally affect bone metabolism, thereby contributing to the onset and advancement of bone-related disorders (Figure 2).

#### Osteoporosis

Osteoporosis is a degenerative bone disorder marked by reduced bone mineral density (BMD), degraded bone microstructure, increased bone fragility, and a higher susceptibility to fractures.^198,199^ Fundamentally, it arises from an imbalance where bone resorption surpasses bone formation, resulting in a loss of bone tissue.^200^ As the population ages, the rates of osteoporosis-related fragility fractures, disability, and mortality are increasing year by year.^201^

**Figure 3.**
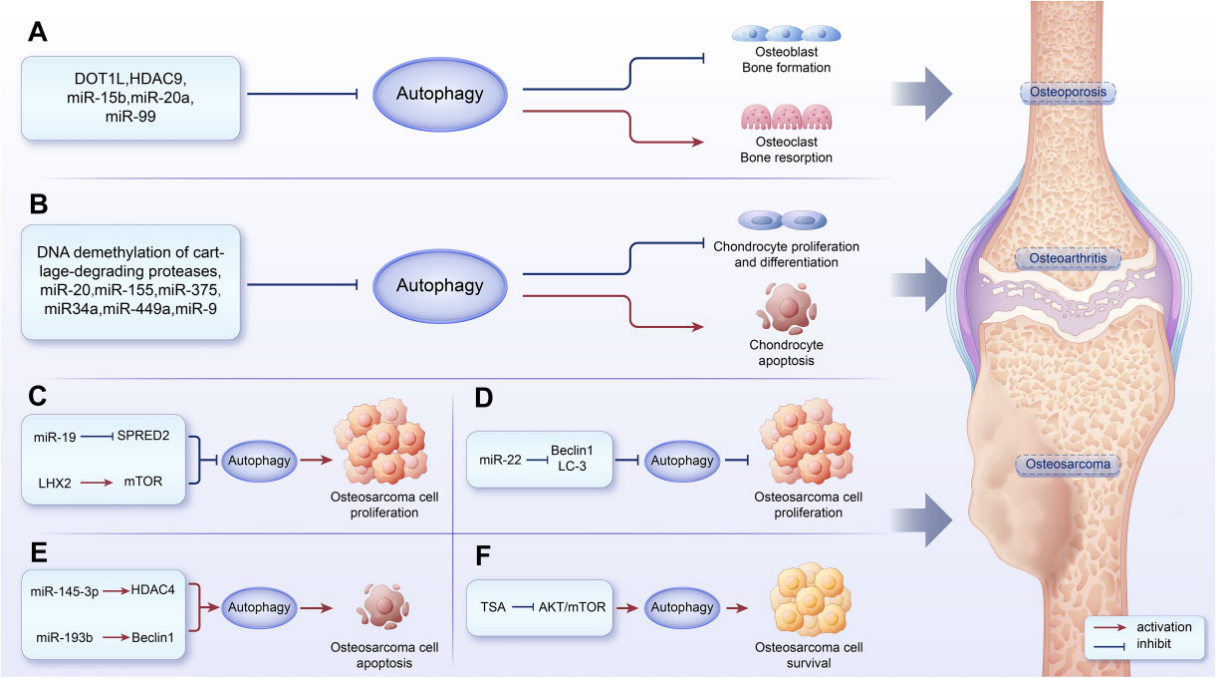
Epigenetic modifications regulate autophagy in bone metabolic diseases. (A) Histone modification and miRNA's inhibition of autophagy results in enhanced osteoclast generation and impedes osteoblast differentiation, causing increased bone resorption relative to bone formation, ultimately leading to osteoporosis. (B) DNA methylation modification and miRNA's inhibition of autophagy lead to increased chondrocyte apoptosis, hindered chondrocyte proliferation, and differentiation, leading to the onset of osteoarthritis. (C-F), Autophagy demonstrates a dual role in osteosarcoma. Its inhibition can either promote or impede the proliferation of osteosarcoma cells. Simultaneously, inducing autophagy can trigger cell apoptosis or elevate the survival rate of osteosarcoma cells.

Numerous studies have confirmed that autophagy increases osteoclast formation under in vitro oxidative stress, low oxygen conditions, and microgravity conditions, leading to an imbalance where bone resorption surpasses bone formation, ultimately triggering osteoporosis (Figure 3).^202,203^ Several studies have demonstrated that miRNAs play a role in regulating bone resorption and bone formation in the process of osteoporosis formation by mediating autophagy-related factors (Figure 2). Research on RAW 264.7 cells under low oxygen conditions revealed that miR-20a directly targets the 3'UTR of ATG16L1, suppressing autophagy by reducing the levels of autophagy-related proteins LC-3 and ATG16L1, which is favorable for osteoclast differentiation. Furthermore, during osteoclast differentiation induced by hypoxia, *HIF-1α* can regulate miR-20a, and the HIF-1α-miR20a-ATG16L1 axis plays a significant role.^204^ MiR-99 may play a role in fine-tuning and integrating the mTOR signaling pathway to promote optimal osteoclast differentiation.^205,206^ Histone demethylases KDM4B and KDM6B play a crucial role in osteogenic commitment of MSCs by removing H3K9me3 and H3K27me3 marks.^207^ Upregulation of USP7 enhances osteogenic differentiation in human adipose-derived stem cells (hASCs) to suppress osteogenesis progression.^208^ USP7 promotes the expression of KDM6B by enhancing its stability.^90^ Elevated levels of miR-15b in osteoporosis inhibit the USP7/KDM6B axis, thereby suppressing osteoblast differentiation and autophagy, exacerbating osteoporosis.^209^ DOT1L, as a histone methyltransferase, can methylate H3k79. Experiments by Gao et al. confirmed that inhibiting DOT1L in vitro increased autophagosome assembly proteins (ATG3 and ATG8-like Gabarapl2) and autophagy receptors (Sqstm1), activated autophagy, increased pre-osteoclast migration, and increased bone density. Absorption increases and osteoporosis occurs.^210^ A research report suggests that age-related upregulation of HDAC9 accelerates bone loss in mice by promoting damaged-induced autophagy.^211^ However, administering an HDAC9 inhibitor to elderly mice can restore mesenchymal osteoblastic function and recover bone mass.^212^

#### Osteoarthritis

Osteoarthritis is a degenerative condition characterized by cartilage degeneration, subchondral bone remodeling, and synovial inflammation. The development of OA is typically associated with factors such as joint stress, genetic predisposition, obesity, aging, and metabolic or hormonal changes.^213^ Additionally, inflammatory mediators such as interleukin (IL)-1, IL-6, and TNF are excessively produced in chondrocytes and matrix cells.^214^ Excessive accumulation of ROS resulting from oxidative stress leads to cartilage degradation.^215^

In the early stages of human OA, the level of autophagy increases, contributing to the maintenance of chondrocyte homeostasis.^216^ However, in late-stage OA, when chondrocytes endure prolonged stress, autophagy weakens, leading to further deterioration of cartilage (Figure 3).^217^

Disruption of normal age-related epigenetic patterns could contribute to age-related conditions such as OA.^218^ Aberrant gene activation in osteoarthritis may be associated with epigenetic derepression that leads to inflammation and catabolic metabolic phenotypes in chondrocytes. Proinflammatory cytokines decrease methylation at crucial CpG sites in the IL-1β promoter, resulting in prolonged induction of this cytokine.^219^ Stimulation of human chondrocytes with IL-1β leads to decreased methylation at CpG sites in the IL-8 promoter and notably triggers this chemokine in chondrocytes affected by OA.^220^ In the study investigating the relationship between autophagy in rat articular chondrocytes and the PI3K/AKT/mTOR signaling pathway in OA, it was found that inflammation can inhibit the proliferation of rat chondrocytes, disrupt the cell cycle, and reduce the rate of autophagy.^221^ Furthermore, DNA demethylation is linked to the upregulation of essential cartilage-degrading proteases such as MMP-3, MMP-9, MMP-13, and ADAMTS-4, as well as iNOS.^222^ Multiple epigenetic modifications have been found to cooperate in regulating gene expression within the context of osteoarthritic lesions. For example, reduced *SOX9* expression in hip OA is likely due to a combination of factors, including elevated DNA methylation, heightened gene-inactivating histone marks (H3K9 and H3K27) methylation, and decreased histone acetylation at the *SOX9* promoter. Furthermore, miRNA-145 has also been confirmed to inhibit the expression of *SOX9*.^223^

Multiple experiments have also confirmed that miRNAs play a significant role in regulating autophagy in the development of OA (Figure 2). MiR-20 is a member of the miR-17-92 cluster located on the chromosome.^224^ In OA, the expression of miR-20 is increased. It can target ATG10 through the PI3K/AKT/mTOR signaling pathway, leading to its reduction, thereby inhibiting chondrocyte proliferation and autophagy.^225^ In the cartilage tissue of OA, miR-375 is found to be overexpressed. It can target the ATG2B–3′ UTR and inhibit its expression in chondrocytes, suppressing autophagy and promoting endoplasmic reticulum stress (ERs), thereby exacerbating cartilage damage.^226^ The experiment indicated that in chondrocytes treated with IL-1β, miR-27a expression is upregulated. It targets the 3′-UTR of the *PI3K* gene, leading to its downregulation. Through the PI3K-AKT-mTOR pathway, miR-27a activates autophagy and inhibits the proliferation of chondrocytes treated with IL-1β, providing a protective effect against OA.^227^ In addition, multiple experiments have confirmed that miR-155,^228^ miR-34a,^229^ and miR-449a^230^ inhibit chondrocyte autophagy, aggravate chondrocyte apoptosis and cause osteoarthritis.

#### Osteosarcoma

Osteosarcoma is a malignant tumor that originates from mesenchymal tissue, typically found at the metaphysis of long bones such as the femur, tibia, and humerus. Although it primarily affects children and adolescents, it can also occur in adults.^231^ The standard treatment for osteosarcoma involves a combination of preoperative and postoperative chemotherapy along with surgical tumor removal, which may even necessitate amputation in severe cases.^66^ Despite aggressive treatment, the prognosis for osteosarcoma remains relatively poor, with a 5-year survival rate of 60%-70% post-surgery. Metastasis, particularly to the lungs, significantly contributes to this unfavorable prognosis, with roughly 20% of patients experiencing metastasis and a subsequent 5-year survival rate of only 30%.^232^

Autophagy plays a dual role at different stages of osteosarcoma (Figure 3). In one study, miR-22 was shown to reduce the expression of Beclin1, LC-3, MTDH, and ATG5 mRNA by targeting MTDH, thereby inhibiting autophagy and suppressing osteosarcoma cell proliferation.^233^ Conversely, inhibiting autophagy was found to promote osteosarcoma cell proliferation in another experiment. In this case, miR-19 was upregulated in osteosarcoma cells, targeting SPRED2 and reducing its expression, which suppressed autophagy, ultimately promoting the proliferation and malignant transformation of osteosarcoma cells.^234^

Epigenetic regulation plays a significant role in autophagy within the context of osteosarcoma (Figure 2). Trichostatin A (TSA), a histone deacetylase inhibitor (HDACi), induces autophagy in osteosarcoma cells by suppressing the AKT-mTOR signaling pathway and activating FoxO1, thereby enhancing the survival of osteosarcoma cells. However, inhibiting autophagy significantly enhances TSA-induced cell death in osteosarcoma.^235^ Additionally, miR-145-3p, which is downregulated in human osteosarcoma cell lines, targets the 3′-UTR region of HDAC4, increasing HDAC4 levels. This, in turn, promotes apoptosis and autophagy in osteosarcoma cells.^236^ Another study involving osteosarcoma cells showed that LHX2 overexpression upregulated mTOR expression, which negatively regulated autophagy through the activation of the mTOR pathway, contributing to the progression of osteosarcoma. MiR-129-5p directly targeted LHX2 3′-UTR to downregulate LHX2, making the miR-129-5p/LHX2/mTOR axis a potential target for osteosarcoma treatment.^237^ Additionally, DANCR is a lncRNA that acts as a ceRNA by sequestering miR-335-5p and miR-1927 in osteosarcoma, promoting ROCK1-mediated proliferation and metastasis.^238^ Another study on osteosarcoma reveals that miR-193b directly targets the 3′-UTR of FEN1, negatively regulating the expression of FEN1, increasing the expression of Beclin 1 and the LC3-II/I ratio, activating autophagy, and inducing cell apoptosis.^239^

#### Rheumatoid Arthritis

Rheumatoid Arthritis (RA) is a chronic autoimmune inflammatory disorder marked by synovial hyperplasia, persistent inflammation, cartilage degradation, and bone erosion.^240–242^ This condition leads to joint deformities and is characterized by swelling and pain. As RA progresses, it can also affect organs and systems outside the joints, such as the heart.^243^ The treatment of RA typically involves a comprehensive approach, including medication, physical therapy, and lifestyle management, aimed at alleviating symptoms, controlling inflammation, and maintaining the patient’s quality of life.^244^ Early diagnosis and treatment are essential for managing the condition and preventing joint damage.^245^

Epigenetic mechanisms, such as DNA demethylation and hypomethylation, play significant roles in altering DNA methylation patterns during B cell to plasma cell differentiation and in RA. These alterations impact disease progression and the expression of key genes.^246,247^ Histone modifications are also involved in RA, with histone H3 in the promoter region of the IL-6 gene being highly acetylated in fibroblast-like synoviocytes (FLS) from RA patients, leading to increased IL-6 expression and disease progression. Inhibitors of HAT, such as curcumin, can reduce IL-6 secretion.^248^

Autophagy in RA has a dual role, exerting both positive and negative effects, depending on specific cellular and molecular regulatory mechanisms.^249,250^ Autophagy plays a crucial role in osteoclast differentiation and maturation under hypoxic conditions, leading to increased bone resorption and accelerated progression of RA.^251^ Additionally, conditions such as nutrient deficiency and endoplasmic reticulum stress promote autophagy, which acts as a self-protective mechanism, allowing RA cells to evade apoptosis and prolong their lifespan.^252^

Research by Li et al. demonstrated that the lncRNA MEG3 is upregulated in synovial tissues of RA patients. MEG3 targets miR-141, and their expression is negatively correlated. By inhibiting the AKT/mTOR pathway and activating autophagy, MEG3 suppresses inflammation, promotes chondrocyte proliferation, and inhibits RA progression.^253^ In experiments by Zhou et al. using a CIA rat model, it was confirmed that treatment with WJR (Wenhua Juanbi Recipe) may inhibit autophagy by affecting the PI3K/AKT/mTOR pathway mediated by miRNA-146a. This inhibition of autophagy leads to the suppression of cell apoptosis and FLS proliferation.^254^ Moreover, in a study by Yang et al., it was found that ZFAS1 (lncRNA ZNFX1 antisense RNA) plays a regulatory role in FLS-RA through the miR-2682-5p/ADAMTS9 axis. Knockdown of ZFAS1 significantly inhibits FLS-RA cell proliferation, inflammatory response, autophagy, and promotes cell apoptosis (Figure 2).^255^

## Future Directions and Challenges

Currently, the group of epigenetic drugs includes^256,257^ DNMTis (such as decitabine used in the treatment of AML and high-risk myelodysplastic syndromes,^258^ and 5-azacitidine used in high-risk myelodysplastic syndromes^259^), HDAC inhibitors (like TSA mentioned earlier, activating autophagy in osteosarcoma^235^), HAT inhibitors (curcumin can reduce IL-6 secretion in RA^248^), histone methyltransferase inhibitors (BIX01294 inhibits G9a in multiple myeloma^260^), and various miRNA-based molecular targeted therapy drugs. Despite their role in disease treatment, these epigenetic drugs lack specificity in target therapy and often exhibit toxicity. Future research directions in the epigenetic regulation of autophagy for bone metabolism-related diseases aim to minimize these drugs’ side effects and identify specific epigenetic modifications involved in autophagy for bone metabolism-related diseases.

The currently well-researched histone modifications in bone metabolism-related diseases include histone methylation and acetylation. Other histone modifications, such as histone phosphorylation,^261^ ADP-ribosylation,^262^ ubiquitination,^263^ SUMOylation,^264^ glutamylation,^265^ glycosylation,^266^ hydroxylation,^267^ and isomerization,^268^ are also covalent modifications that potentially regulate gene expression by altering chromatin structural states and functions, or affecting the affinity between transcription factors and gene promoters. Despite extensive research in fields such as tumorigenesis, energy metabolism, and cellular aging, limited reporting exists on their role in bone metabolism-related diseases. Future comprehensive investigations into the regulation of these histone modifications in autophagy concerning bone metabolism-related diseases might unveil new targets for preventing and treating these conditions and guide the development of novel epigenetic drugs.

Research on miRNA regulation of autophagy in bone metabolism-related diseases is burgeoning and provides potential targets for treating these conditions. A crucial aspect in precise treatment for bone-related diseases involves transferring target miRNAs or anti-miRNAs to target cells without being degraded by endogenous RNA or causing off-target effects. Presently, experimental delivery systems include aptamers, single-stranded DNA or RNA,^269,270^ 8-repeat aspartic acid sequences (D-Asp8),^271^ and bacteriophage MS2 virus-like particles (MS2 VLPs).^272^ Future advancements should emphasize developing delivery systems that enhance miRNA stability and cellular uptake efficiency. MiRNAs, capable of mimicking or inhibiting the expression of target genes, have the potential to regulate bone biology processes such as bone formation, resorption, and regeneration by activating or inhibiting autophagy.

## Conclusion

In this paper, we delved into the critical roles of genetic regulation and autophagy in bone metabolism and highlighted key insights relevant to bone health. Through the culmination of our research, we draw the following conclusions:

Firstly, genetic regulation and autophagy play pivotal roles in bone metabolism. Genetic regulation, by modulating gene expression, determines the differentiation, proliferation, and function of bone cells. Autophagy, on the other hand, is a self-regulating cellular process indispensable for clearing aged or damaged cells, maintaining intracellular homeostasis, and facilitating bone tissue repair. These two mechanisms intricately intertwine and cooperate to uphold bone health.

Secondly, research indicates that abnormalities in genetic regulation and autophagy are closely associated with the onset and progression of various bone metabolic disorders and bone-related diseases. Not only do genetic mutations lead to the hereditary transmission of certain bone diseases, but environmental factors, lifestyles, and aging impact the functionality of autophagy, thereby exerting adverse effects on bone health. Consequently, understanding the roles and dysregulation of these regulatory mechanisms is paramount for preventing, diagnosing, and treating bone disorders.

Lastly, we emphasize the significance of further research in propelling advancements in the field of bone health. By delving deeper into the molecular mechanisms of epigenetic regulation and autophagy, we can identify novel therapeutic targets and develop more effective treatment strategies. Simultaneously, with the aid of precision medicine and personalized treatment approaches, we can better cater to the diverse needs of individual patients, elevating the management of bone diseases.

In conclusion, this paper underscores the importance of genetic regulation and autophagy in maintaining bone health and their close connections with bone metabolic disorders and related diseases. We encourage future research to continue delving into these domains, with the aim of providing innovative solutions for preventing and treating bone disorders, ultimately enhancing the quality of life and health of patients. Through collaboration and exploration of new frontiers, we can collectively drive progress in the field of bone health, paving the way for new possibilities in future medical and clinical practices.

## Data Availability

The data involved in this review article are available from the corresponding author on reasonable request.
